# Spotted Fever Group Rickettsiae in Inner Mongolia, China, 2015–2016

**DOI:** 10.3201/eid2411.162094

**Published:** 2018-11

**Authors:** Gaowa, Wulantuya, Xuhong Yin, Shengchun Guo, Chunlian Ding, Minzhi Cao, Hiroki Kawabata, Kozue Sato, Shuji Ando, Hiromi Fujita, Fumihiko Kawamori, Hongru Su, Masahiko Shimada, Yuko Shimamura, Shuichi Masuda, Norio Ohashi

**Affiliations:** College of Hetao, Bayan Nur City, Inner Mongolia, China (Gaowa, Wulantuya, X. Yin, S. Guo, C. Ding);; Bayan Nur Centers for Disease Control and Prevention, Bayan Nur City (M. Cao);; National Institute of Infectious Diseases, Shinjuku-ku, Tokyo, Japan (H. Kawabata, K. Sato, S. Ando);; Mahara Institute of Medical Acarology, Anan City, Tokushima, Japan (H. Fujita);; University of Shizuoka, Shizuoka City, Japan (F. Kawamori, H. Su, M. Shimada, Y. Shimamura, S. Masuda, N. Ohashi)

**Keywords:** *Rickettsia*, *Rickettsia raoultii*, *Rickettsia aeschlimannii*, *Hyalomma asiaticum*, *Dermacentor nuttalli*, *Hyalomma marginatum*, spotted fever group rickettsiae, *gltA*, 16S rDNA, human infection, vector-borne infections, Inner Mongolia, China, bacteria, ticks

## Abstract

We found *Rickettsia raoultii* infection in 6/261 brucellosis-negative patients with fever of unknown origin in brucellosis-endemic Inner Mongolia, China. We further identified *Hyalomma asiaticum* ticks associated with *R. raoultii*, *H. marginatum* ticks associated with *R. aeschlimannii*, and *Dermacentor nuttalli* ticks associated with both rickettsiae species in the autonomous region.

Spotted fever group rickettsiae (SFGR) are vectorborne pathogens. In China, 5 SFGR genotypes have been identified as causative agents of human rickettsiosis: *R. heilongjiangensis*, *R. sibirica* subsp. *sibirica* BJ-90, *Candidatus* Rickettsia tarasevichiae, *R. raoultii*, and *Rickettsia* sp. XY99 (*1**–**4*). 

Brucellosis, a zoonotic disease, is highly endemic to Inner Mongolia, China, and is increasing in workers in agriculture or animal husbandry (*5*). However, some agriculture workers with brucellosis-like symptoms, including general malaise and fever, were seronegative for *Brucella* spp. We suspected that fever of unknown origin among brucellosis-seronegative patients might be caused by tickborne pathogens. We identified 6 cases of human *R. raoultii* infections in brucellosis-seronegative patients in western Inner Mongolia, and we investigated exposure to ticks infected with SFGR.

During 2015–2016, we obtained 261 blood samples from brucellosis-seronegative patients with fever of unknown origin in Bayan Nur Centers for Disease Control and Prevention (Bayan Nur City, Inner Mongolia, China). The review board of the Department of Medicine at College of Hetao (Bayan Nur City) approved the study. We extracted DNA from each blood sample using the DNeasy Mini Kit (QIAGEN, Hilden, Germany) and conducted PCR targeting SFGR *gltA* (*6*). The PCR primers used, gltA-Fc (5′-CGAACTTACCGCTATTAGAATG-3′) and gltA-Rc (5′-CTTTAAGAGCGATAGCTTCAAG-3′), were described previously (*4*). We designed the primers 16S rDNA R-2F (5′-GAAGATTCTCTTTCGGTTTCGC-3′), 16S rDNA R-2R (5′-GTCTTGCTTCCCTCTGTAAAC-3′), rompA-Fb (5′-GGTGCGAATATAGACCCTGA-3′), and rompA-Ra (5′-TTAGCTTCAGAGCCTGACCA-3′) for this study and deposited the sequences obtained of *gltA*, *ompA*, and 16S rDNA into GenBank (accession nos. MH267733–47). We used genomic DNA extracted from L929 cells infected with *Rickettsia* sp. LON-13 (*gltA*: AB516964) as a positive control. 

We detected *gltA* amplicons from 6/261 (2.3%) blood samples (Table). All 6 patients had strong malaise and mild fever of 36.8°C –37.3°C but no rash. Five of these patients also had arthralgia and vomiting.

**Table:** PCR survey of SFGR infections in patients and ticks, Inner Mongolia, China, 2015–2016*

Patient type or tick species	No. tested	No. (%) *gltA* positive for SFGR		
*Rickettsia raoultii*	*R. aeschlimannii*	Total
Brucellosis-seronegative patients	261	6 (2.3)	0	6 (2.3)
Ticks				
* Hyalomma asiaticum*	766	118 (15.4)	0	118 (15.4)
* Hyalomma marginatum*	198	0	160 (80.8)	160 (80.8)
* Dermacentor nuttalli*	1,418	830 (58.5)	158 (11.1)	988 (69.7)†
* Rhipicephalus turanicus*	76	0	0	0
Total ticks	2,458	948 (38.6)	318 (12.9)	1,266 (51.5)

*SFGR, spotted fever group rickettsiae. †We did not detect dual infection with *R. raoultii* and *R. aeschlimannii* in *D. nuttalli* ticks in this study.

Sequence and phylogenetic analysis showed that the sequences of 6 nearly full-length (1.1 kb) *gltA* amplicons with were identical to each other and to *R. raoultii gltA* (GenBank accession no. DQ365803). We further analyzed *ompA* and 16S rDNA in *gltA*-positive samples. All 6 samples were PCR positive for both genes; 552-bp sequences of the amplicons were identical to sequences of *R. raoultii ompA* (GenBank accession no. AH015610), and 389-bp sequences of the amplicons were identical to sequences of *R. raoultii* 16S rDNA (GenBank accession no. EU036982). PCR results were negative for the genes *Anaplasma phagocytophilum p44*/*msp2*, *Ehrlichia chaffeensis p28*/*omp-1*, and *Borrelia* spp. *flaB*. An indirect immunofluorescence assay showed that IgM and IgG titers against *R. japonica* were 40–80 for IgM in 3 patients and 160 for IgG in 2 patients. 

To assess patients’ risk of infection with SFGR by tick exposure, we collected 2,458 ticks morphologically identified as *Hyalomma marginatum* (n = 198), *H. asiaticum* (n = 766), *Dermacentor nuttalli* (n = 1,418), and *Rhipicephalus turanicus* (n = 76) from livestock and pet animals including sheep, cattle, camels, and dogs in western Inner Mongolia during 2015–2016 (Technical Appendix Figure 1). We collected unattached ticks within animal hair, but not attached ticks. We prepared DNA extracted from salivary glands of each tick and conducted PCR screening by rickettsial *gltA* detection as described. We detected *gltA* in 1,266 (51.5%) of the total 2,458 ticks. 

We classified the amplicons into 2 groups by restriction fragment-length polymorphism using *Alu*I and *Rsa*I, and we sequenced 25–45 representative amplicons in each group. On the basis of this analysis, we found that the sequences from the 2 groups were either identical to that of *R. raoultii* (GenBank accession no. DQ365803) or to that of *R. aeschlimannii* (GenBank accession no. HM050276) (Table; Technical Appendix Figure 2). We detected *R. raoultii* DNA in *H. asiaticum* (118/766, 15.4%) and *D. nuttalli* (830/1,418, 58.5%) ticks and *R. aeschlimannii* DNA from *H. marginatum* (160/198, 80.8%) and *D. nuttalli* (158/1,418, 11.1%) ticks. We did not detect rickettsial DNA in *R. turanicus* ticks (0/76, 0%).

Recently, human cases of *R. raoultii* infection have been reported in China, including northeastern Inner Mongolia (*1**,**4*). Potential vectors for *R. raoultii* are *Dermacentor* spp. ticks in Europe, Turkey, and northern Asia and *Haemaphysalis* spp. and *Amblyomma* sp. ticks in southern Asia (*7**,**8*). Other studies have identified *Hyalomma* spp., *Rhipicephalus* spp., and *Amblyomma* sp. ticks as potential vectors for *R. aeschlimannii* (*7**,**8*); human cases of *R. aeschlimannii* infection have been reported in Italy and Morocco (*7**,**9*). We detected *R. raoultii* in *H. asiaticum* as well as *D. nuttalli* ticks, but in Mongolia, *R. raoultii* has been detected only in *D. nuttalli* ticks, and not *H. asiaticum* ticks (*10*). We identified *D. nuttalli* ticks as another potential vector for *R. aeschlimannii*. Our work contributes to the knowledge of the epidemiology, clinical characteristics, and known tick vectors associated with *R. raoultii* and *R. aeschlimannii*.

Technical Appendix Additional information about spotted fever group rickettsiae in Inner Mongolia, China.
