# Supplementary material for: Spotted Fever Group Rickettsiae in Inner Mongolia, China, 2015–2016
Source: Emerg Infect Dis. 2018 Nov;24(11):2105–7. doi: 10.3201/eid2411.162094 (PMC6200000; doi:10.3201/eid2411.162094)
Supplement: Technical Appendix — Additional information about spotted fever group rickettsiae in Inner Mongolia, China. [file 16-2094-Techapp-s1.pdf]

Article DOI: https://doi.org/10.3201/eid2411.162094

Spotted Fever Group Rickettsiae in Inner Mongolia, China, 2015–2016

Technical Appendix

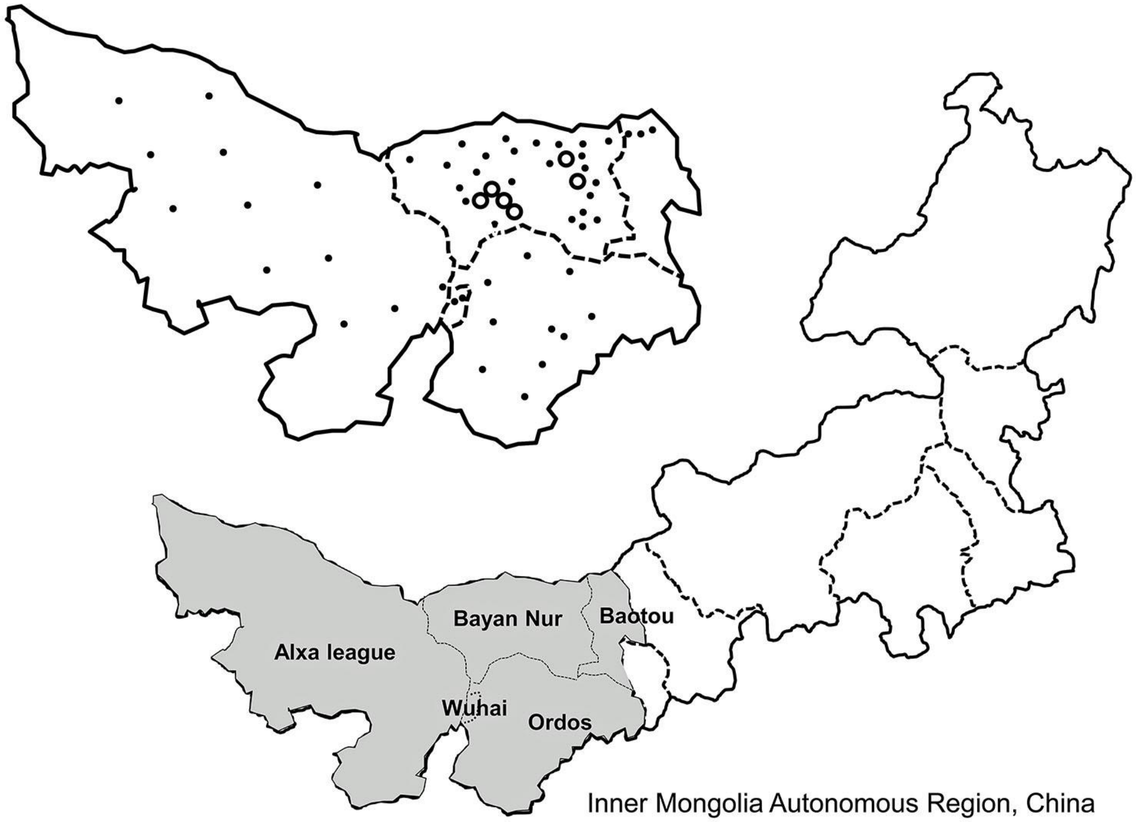

Technical Appendix Figure 1. Locations of 6 patients with *Rickettsia raoultii* infection (open circles) and tick collection sites (solid dots) in western Inner Mongolia. Tick species collected were morphologically identified as *Hyalomma marginatum, H. asiaticum, Dermacentor nuttalli,* and *Rhipicephalus turanicus* that are known to be distributed in Asia, including Mongolia, and Turkey (*1–3*).

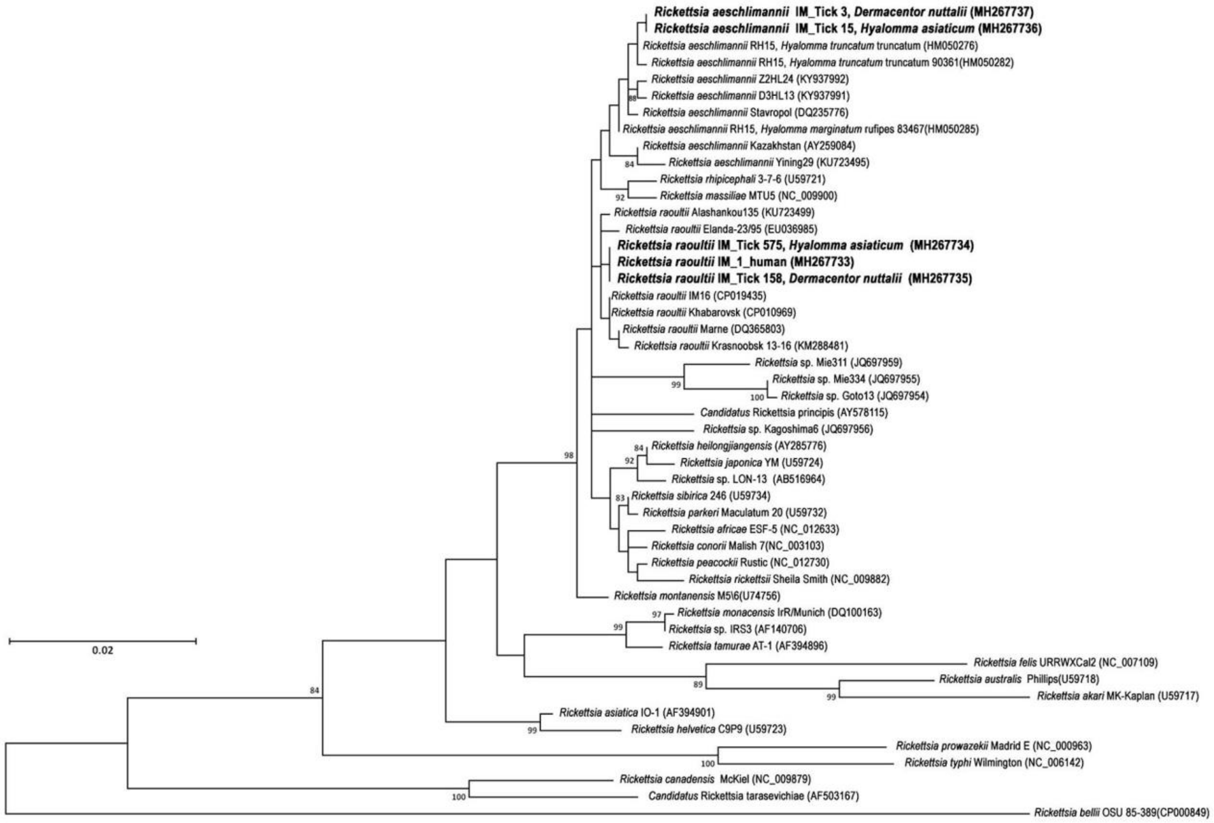

Technical Appendix Figure 2. Phylogenetic tree for spotted fever group rickettsiae based on *gltA* sequences (1,017 bp) in patients and in ticks in western Inner Mongolia, China. The tree was constructed using the maximum-likelihood method with 100 bootstrap resamplings in MEGA 6 (www.megasoftware.net). Bold indicates spotted fever group Rickettsia detected in this study. Scale bar indicates evolutionary distance.

References

1. Parola P, Paddock CD, Socolovschi C, Labruna MB, Mediannikov O, Kernif T, et al. Update on tick-borne rickettsioses around the world: a geographic approach. Clin Microbiol Rev. 2013;26:657–702. 1 PubMed http://dx.doi.org/10.1128/CMR.00032-13

2. Karasartova D, Gureser AS, Gokce T, Celebi B, Yapar D, Keskin A, et al. Bacterial and protozoal pathogens found in ticks collected from humans in Corum province of Turkey. PLoS Negl Trop Dis. 2018;12:e0006395. PubMed http://dx.doi.org/10.1371/journal.pntd.0006395

3. Boldbaatar B, Jiang RR, von Fricken ME, Lkhagvatseren S, Nymadawa P, Baigalmaa B, et al. Distribution and molecular characteristics of rickettsiae found in ticks across Central Mongolia. Parasit Vectors. 2017;10:61. PubMed http://dx.doi.org/10.1186/s13071-017-1981-3
